# Return to the hematopoietic stem cell origin

**DOI:** 10.1186/2045-9769-1-9

**Published:** 2012-12-20

**Authors:** Igor M Samokhvalov

**Affiliations:** South China Institute of Stem Cell and Regenerative Medicine, Guangzhou Institutes of Biomedicine and Health, Chinese Academy of Sciences, Guangzhou, 510530 China

**Keywords:** Hematopoiesis, Development, Hematopoietic stem cell, Repopulation, Cell potential, Cell tracing

## Abstract

Studying embryonic hematopoiesis is complicated by diversity of its locations in the constantly changing anatomy and by the mobility of blood cell precursors. Embryonic hematopoietic progenitors are identified in traditional *in vivo* and *in vitro* cell potential assays. Profound epigenetic plasticity of mammalian embryonic cells combined with significant inductive capacity of the potential assays suggest that our understanding of hematopoietic ontogenesis is substantially distorted. Non-invasive *in vivo* cell tracing methodology offers a better insight into complex processes of blood cell specification. In contrast to the widely accepted view based on the cell potential assays, the genetic tracing approach identified the yolk sac as the source of adult hematopoietic stem cell lineage. Realistic knowledge of the blood origin is critical for safe and efficient recapitulation of hematopoietic development in culture.

## Introduction

All blood cells of adult organism are produced in the process of hematopoiesis which depends entirely on the ability of hematopoietic stem cells (HSCs) to self-renew and differentiate into all hematopoietic cell lineages. In mice, the whole population of adult HSCs is formed during embryogenesis and first neonatal weeks [1]. This process has been described in a number of detailed reviews which reflect the current understanding of the HSC development [2–5]. However, a closer look at experimental data reveals significant contradictions in the current paradigm of hematopoietic ontogenesis, and demands reassessment in view of the recent findings [6–8]. This is especially important if the lessons learned from studying the HSC development are meant to be used for future clinical applications. This review is an attempt of alternative interpretation of published experimental data.

Early mammalian development is highly regulative [9] and it is difficult to determine how long this capacity is operational in early ontogenesis. Isolated parts or tissues of precirculation conceptuses have the ability to adapt to the changed microenvironment by switching to an alternative developmental pathway tissues. This capacity therefore substantially complicates finding the anatomical origin of mammalian hematopoiesis by standard hematological approaches. However, practically all information about development of hematopoietic progenitors and HSCs is based on the analysis of embryonic cell potentials in a number of *in vivo* and *ex vivo* assays. Accumulating evidences on the extent of cell plasticity [10–13] and striking ease of cell reprogramming [14–16] suggest that there might be a significant gap between measured potential of a cell and its fate in developing conceptus. The assays may lead to artefactual inductions that change initial commitment of a cell and promote specification in an alternative direction. Moreover, the assays can selectively kill certain subsets of hematopoietic progenitors by imposing a strong non-physiological stress. Mere dissociation of embryonic tissue before the assay can induce profound changes in cell behavior [17]. Due to their unsettled epigenetic status embryonic cells are more vulnerable than adult cells to various inducing events during the potential assays. And from a practical point of view, it is difficult to detect the induced cell plasticity event in embryonic system due to the absence of defined starting cell types.

Another critical problem is that analyzing cell potentiality is often skewed towards conditionally pluripotent or highly multipotent cells which may be present in conceptus tissues. These cells can give rise to hematopoietic progeny in a process similar to embryonic stem (ES) cell differentiation, and the term of the hematopoietic progenitor or even the HSC might be falsely awarded to a cell which is not committed to differentiate into blood *in vivo*.

For molecular studies, the classical hematological assays are valuable since they outline the emerging potentials during progressive mesoderm specification. However, the cell potential dynamics are unlikely to accurately describe developmental processes. The potential data may prove misleading if we are to recapitulate *in vitro* the natural sequence of embryonic cell inductions and maturations from such epiblast cell surrogates as ES or iPS cells.

### Terms and definitions

Controversy surrounding the issue of mammalian blood origin to a large extent results from the confusion with the understanding and interpretation of basic terms and definitions. One of these, “definitive HSC”, has to designate cells which self-renew and produce committed hematopoietic progenitors at a suitable site of hematopoiesis. Definitive HSCs (dHSCs) are detected using their ability to serially repopulate normal, i.e. non-immunodeficient or otherwise genetically compromised, myeloablated recipients. However, it remains an open issue that some fetal cells may display the HSC potential even in a stringent repopulation assay but do not function as dHSCs *in situ*. It has been difficult to determine when dHSCs start to generate blood lineages in conceptus due to retrospective way of the HSC definition. In the repopulation assay tested cells are introduced into the severely conditioned environment of mouse recipient and their progeny is traced for several months to confirm the long-term repopulation potential. It is possible that apparent stem cell potential of a tested cell population is actually induced within the recipient hematopoietic system and a few newly generated HSCs are efficiently selected for survival. First dHSC potential is found at embryonic day 10.5-11.0 (E10.5-E11.0), when the low-level adult-recipient repopulating activity is first detected in the aorta-gonads-mesonephros (AGM) region [18]. However, there is no evidence that these cells actually function as typical HSCs at this stage *in vivo*, i.e. self-renew and clonally differentiate into a number of blood lineages. On the contrary, blood cell generation does not occur [19, 20], and cell proliferation in general has been found to be very low in the AGM region at this stage [21]. At E11.5-12.5 dHSCs expand in fetal liver and possibly in placenta. Again, we can say only about the expansion of a potential, but not about the expansion of the stem cell function. The fetal liver hematopoiesis (mainly erythropoiesis) is substantially increased at this stage, although this might be accomplished mainly by unipotent or oligopotent progenitors lacking the crucial self-renewal capacity [2].

To circumvent the difficulty with staging the onset of dHSC function, the term “HSC lineage” can be introduced or redefined. In developmental hematology this term describes all developmental history of strictly defined dHSCs starting from the earliest mesodermal precursor cells. Since early mammalian development is highly regulative it is necessary to exclude the zygote, blastomeres, inner cell mass (ICM) and epiblast cells from the HSC lineage. The lineage includes committed toward hematopoiesis mesodermal cells in early embryo, their progeny leading eventually to mature stem cells and which can be called pre-HSCs (intermediate cells ultimately giving rise to the initial dHSC population) and two major subtypes – fetal and adult – of dHSCs. Many members of the HSC lineage do not possess the HSC potential and cannot function as stem cells; they have to undergo specification, selection and maturation to become fully operative dHSCs. In the conceptus this lineage coexists with others, such as primitive erythroblasts, macrophages and megakaryocytes, and a number of definitive transient hematopoietic progenitor lineages in yolk sac, fetal liver and other fetal hematopoietic organs constituting the first wave of definitive hematopoiesis [22]. The relationship between all these conceptal hematopoietic cell lineages is unclear, though recent cell tracing experiments point to the probability that all these lineages have common mesodermal precursors [6, 8].

Another specific term which needs clarification is “*de novo* hematopoiesis”. The purpose of the term is to distinguish two distinct processes of blood cell formation: the hematopoiesis itself, i.e. generation of blood cells from an existing hematopoietic precursor, and differentiation of the lateral mesoderm into first cells which can be regarded as belonging to a blood cell lineage. The obvious difficulty in the definition is the uncertainty about criteria which can be used to define emerging cells as hematopoietic. Perhaps induction of molecular signatures similar to the early hematopoietic triade [23] can be chosen to distinguish this type of hematopoiesis. The term reflects an objective process of primary blood generation in the conceptus, whereas the expansion and maturation of newly formed hematopoietic precursors can be defined as secondary developmental hematopoiesis which is distinct from the “classical” hematopoiesis initiated by mature hematopoietic progenitors or dHSCs. *De novo* hematopoiesis can be regarded as segregation of blood-committed cells from other mesodermal precursors. By definition, *de novo* hematopoiesis is always an autonomous process, whereas the secondary and “the classical” types of hematopoiesis are mostly non-autonomous and depend on immigration of progenitors from the sites of the primary or *de novo* hematopoiesis.

### Inadequate methodology

What an undifferentiated cell can do in an unnatural environment seems to depend entirely on the conditions used in an assay. A typical example is so-called random commitment of adult HSCs in methylcellulose (Mtc) cultures [24, 25]. When challenged *in vitro* by a combination of exogenous cytokines, freshly isolated HSCs that are capable of long-term multilineage repopulation of the recipient's hematopoietic system can spontaneously and randomly differentiate into lineage–committed hematopoietic progenitors and generate corresponding mature blood cells. Even highly purified HSCs easily form colonies of differentiated hematopoietic cells both in liquid and methylcellulose media supplemented with hematopoietic cytokines. Evidently, isolation of the stem cells from their native niche removes any inhibitory signaling and restores their capacity to respond to proliferation/differentiation cues. More than 90% of single CD150^+^CD48^─^CD41^─^c-kit^+^Sca-1^+^Lin^─^ cells, which are essentially pure HSCs (1 in 3 cells gave long-term multilineage reconstitution) [26], formed mainly CFU-mix (colony forming unit – mixed) and CFU-GM (colony forming unit – granulocyte/macrophage) in a standard Mtc assay [26, 27]. Around 80% of CD34^─^KSL cells gave rise to colonies of differentiated hematopoietic cells in the liquid cultures supplemented with cytokines [28].

The potential of many early conceptus cells is essentially conditional. E9.0 yolk sac cells do not repopulate conditioned adult bone marrow, whereas they become functional after injection into conditioned newborn fetal liver [29, 30]. Cells from the E8.0-E8.5 yolk sac were by no means capable of rescuing hematopoiesis in irradiated adult recipients, but they gained the long-term engraftment activity after co-culture with the stromal cell line derived from the AGM region [31]. Cells freshly isolated from the paraaortic splanchnopleura (P-Sp) could not directly engraft the bone marrow of adult immunodeficient recipients, but they get a chance when cultured within the explant for several days [32, 33]. Clearly, in the search for hematopoietic progenitors their putative precursors are often forced to undergo additional development. Could it occur also in the standard hematological assays?

There is direct experimental evidence of the gap between measured cell potential and the actual function of the hematopoietic precursors in developing embryo. Analyzed in an *in vitro* assay the hemangioblast potential was found mostly (75%) around the primitive streak [34] at the stage at which the mesodermal masses in the proximal yolk sac were clearly committed to hematopoiesis and largely segregated from endothelial precursors located more distally in the yolk sac [35, 36]. Cell tracing experiments showed the absence of any perceptible hemangioblasts activity *in vivo* or in the cultured conceptus [6, 37, 38]. Apparently, when mesodermal cells are subjected to high concentration of VEGF and c-Kit ligand in culture, they generate cell colonies capable to differentiate into endothelial and blood cells, but in reality these two cell lineages segregate immediately after the emergence from the primitive streak. Differentiated definitive erythroid progenitors (CFU-E, colony forming units – erythroid) can be detected in the yolk sac already at E8.5 by standard methylcellulose assay [39]. The presence of these progenitors is a clear sign of terminal stages of active erythropoiesis, but the first mature definitive erythrocytes are detected in mouse embryo almost 4 days later [40, 41]. The most feasible explanation is that some immature hematopoietic precursor cells which are not actually producing mature blood cells can nevertheless survive in the assay and differentiate into CFU-Es. These precursors in real development would need the environment of fetal liver to develop into the erythroid progenitors and possibly into other hematopoietic progenitors including HSCs.

The mentioned above notion of the absence of active hematopoiesis in the AGM region highlights the difference between the HSC potential and the HSC function. Fetal HSCs are known to actively proliferate [1] and normally stem cell proliferation is accompanied by their differentiation into committed hematopoietic progenitors. The failure of hematopoietic progenitor generation in the AGM region was explained by incompatibility of *de novo* generation of dHSCs with the secondary hematopoiesis [19]. Alternatively, the AGM cells that display stem cell potential in conditioned adult recipients may not be actually capable of performing the HSC function in embryo, but rather have reached an epigenetic status compatible with adult bone marrow inductive environment which allows them to survive and produce differentiated multilineage progeny. It is possible that only a very small fraction of the transplanted cell population is selected to survive and adapt to assay’s microenvironment. Earlier precursor cells may be not prepared yet to survive the assays environments because they are not capable yet to recognize the maintenance or survival signals. The maturation, or directed epigenetic changes, would make them more suitable for the assays’ challenges. In this view, the potential assays do not study the progenitor dynamics, instead they expose the sequence of epigenetic changes in the developing immature precursor cells which cannot be characterized yet as hematopoietic progenitors or stem cells.

The developmental hematology thus has to concentrate on discerning the “cellular pathways” which start from the emergence of lateral mesoderm and culminate in the formation of functional *in situ* dHSCs. The pathways have to be revealed through the analysis of the crosstalk between particular phenotypic and epigenetic state of the hematopoietic cells and corresponding inductive environments in the conceptus. An appropriate methodology for this is genetic cell tracing analysis performed *in vivo,* or in the long-term culture of midgestation mouse conceptuses, or during *in vitro* differentiation of human pluripotents cells.

### Problems with intraembryonic sites of *de novo* hematopoiesis

The central role in the HSC ontogenesis has been ascribed to the paraaortic splanchnopleura (P-Sp) which later transforms into the AGM region [42]. It is generally thought that the intra-embryonic HSCs develop in close association with the ventral wall of the dorsal aorta, and perhaps with the endothelium of vitelline/umbilical arteries and placental vessels. This *de novo* hematopoiesis is believed to happen in hematopoietic intra-aortic clusters (HIACs) which are seen to be attached to the endothelium of big vessels during midgestation in mice and 5th week of gestation in human. Direct involvement of endothelial cells in the process has been postulated [43], and the concept of hemogenic endothelium has been developed [44, 45]. In a slightly different view, the autonomous source of the HSC lineage lies beneath dorsal aorta in subjacent mesenchyme [46], or in subaortic patches [47], so that developing pre-HSCs migrate towards lumen of aorta and eventually formed hemogenic clusters. The hemogenic endothelium hypothesis is currently getting wider support [4] though there is probably no big difference between these two models. The ancestral P-Sp region was reported to contain some cells of the HSC lineage, which seem to arise there autonomously. These cells are believed to become committed to develop into cells with some repopulation potential [32, 33] at the time when aortic vasculogenesis takes place [40]. This means that some mesodermal precursors belonging to the HSC lineage could perform the incorporation into the endothelial layers of the aorta, and then would “transdifferentiate” into HIACs. However, this transdifferentiation would happen at the moment when dorsal aorta and other major vessels sustain a vigorous extension and remodeling in addition to the induction of intensive angiogenesis into adjacent tissues [48]. It might be possible that cell clusters forming inside of big vessels, instead of being a product of local proliferation of hemogenic endothelium, are in fact involved in the development of vascular system. Indeed, endothelial cell proliferation in the floor of midgestation dorsal aorta was reported to be minimal if not completely absent [49]. The emergence of HIACs in zebrafish was recently suggested to be associated with vascular remodeling [50], and it still remains to be demonstrated whether HIACs contain cells with the dHSC potential.

There are two major problems with depicting the P-Sp as an early source of the HSC lineage. First, there is a substantial risk of primordial germ cells (PGCs) being involved in the manifestation of progenitor or stem cell activity. These cells at E8.5-E9.0 are located in the caudal part of P-Sp (where most of blood clonogenic activity was detected [32]) being on the migration route from mesodermal layer into definitive endoderm of primitive gut [51, 52]. The PGCs are conditionally pluripotent cells and have potential to differentiate *in vitro* into multiple hematopoietic lineages [53, 54]. One cannot therefore exclude a possibility that PGCs can display a HSC potential after being introduced into conditioned immunodeficient recipient. It is probably not a simple coincidence that PGCs arrive into the AGM region between E10.5-E11.0 [55] when the first HSC potential is detected there. Similar to primitive blood cells the PGCs originate in extraembryonic mesoderm and share with developing hematopoietic cells the expression of such markers as c-Kit [51], CD31 (PECAM) [56, 57], integrin β1 [58] and CXCR4 [59]. It is unclear to which extent the HSC potential can be attributed to PGC contamination in the E10.5-E11.0 AGM region since it is difficult to reliably separate HSC and PGC lineages at this stage.

There are other cells in early embryo which could develop HSC potential in hematopoietic assays. Mouse embryo gastrulation starts at E6.5 and continues until around E10.5, when caudal neuropore is finally closed [60]. It is unclear whether primitive streak cells are capable to display a hematopoietic potential in hematological assays, but it is feasible that cells newly derived from epiblast may be prone to show a significant multipotentiality, even though they has been characterized as “committed” to one or another cell fate [61, 62]. Primitive streak cells are also located in the caudal part of P-Sp and it is very difficult to separate them from the lateral mesoderm layer in the P-Sp explants. They might therefore contribute to the HSC/progenitor potential of intraembryonic cells.

The pivotal experiments supporting the AGM’s role as an autonomous source of dHSCs includes short-term *ex vivo* organ culture of the AGM, fetal liver and the yolk sac [63]. The AGM showed an extraordinary propensity for the expansion of the E10.5-E11.0 dHSC potential during the explant culture compared to fetal liver and yolk sac. Since the AGM microenvironment is not destroyed in the briefly cultured explant, one can think that most of HSC-inducing signals are preserved, creating appropriate conditions for accumulation of stem cell potential. This was the reason to suggest that the region was an autonomous intraembryonic site of dHSCs *de novo* generation. However, when an early E9.5 P-Sp/AGM was subjected to the explant culture no accumulation of the stem cell potential was observed [64]. If the AGM region is the autonomous source of HSCs, it would be reasonable to assume that the E9.5 explants should also generate dHSC potential in the same culture conditions. A number of plausible explanations can be put forward to explain the absence of the potential at the end of the E9.5 AGM explant culture, but if the “nascent pre-HSCs” are already present in the ancestral E8.0 P-Sp region [33], it would be difficult to accept them without reservations. The simplest and therefore most plausible explanation (apart from the PGC involvement) is that the stem cell precursors are not yet present in the AGM region before E9.5 but appear there later, in the period between E9.5 and E10.5, most likely immigrating from the yolk sac. This would explain why yolk sac does not accumulate the stem cell potential in the explant cultures [63, 64]: pre-HSCs are leaving the yolk sac environment probably because it is not suitable for induction or maintenance of the dHSC potential [36].

If dHSCs emerge from the floor of the dorsal aorta, they would require some local inductive signals emanating from adjacent intraembryonic tissues. However, HIAC-like cell clusters presumably possessing the dHSC potential appear at the same stages in umbilical and vitelline arteries [49, 65–67]. Even though the vessels are directly connected to the dorsal aorta, they do not have the same potentially inductive tissues nearby. This would invoke two different mechanisms of *de novo* generation of dHSCs, unless HIAC cells can dissociate from the clusters, “roll” along the vasculature and re-attach to other endothelial beds.

### The yolk sac reloaded

A number of recent publications may change the current understanding of developmental hematopoiesis [6–8]. The rodent visceral yolk sac is a very prominent part of the conceptus and provides mechanical protection to the fetus at later stages of development and birth. Early in development, immediately after its formation, the visceral yolk sac serves as a primitive placenta for the short period of time [68]. The early yolk sac is formed by essentially simple bilaminar tissue consisting of extraembryonic lateral mesoderm and an adjacent layer of primitive visceral endoderm [35]. The yolk sac is quickly vascularized, and initial simple vascular plexus becomes fused with the embryo vasculature at the onset of blood circulation. When the circulation starts at E8.25-E8.5, the yolk sac vasculature begins to be remodeled eventually forming the yolk sac vascular system.

**Figure 1:**
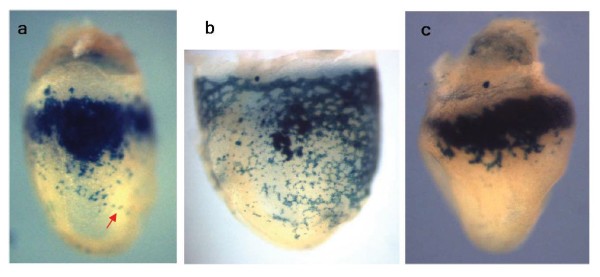
**Yolk sac blood cells at E8.25-E8.5. Hematopoietic cells were visualized by Runx1/LacZ staining.** (**a**) “Blood island” - like cell aggregations appear in the proximal yolk sac at around the start of blood circulation. The red arrow points to blood cells on their way through vitelline vein into embryonic vasculature. (**b**) Dense cell clusters in the proximal region of the yolk sac resist the circulation drive for some period of time. (**c**) In pre-circulation conceptus blood cells start to move distally before the yolk sac vascular plexus fuses with the embryo vasculature.

During gastrulation the extraembryonic mesoderm moves towards the ectoplacental cone and builds up significant cell masses in the proximal yolk sac region at around E7.5. These mesodermal cell masses accumulate globin during the day 9 of gestation (E8.0-E9.0) and develop into structures which are called the “blood islands” which look like reddish spots in the proximal part of dissected yolk sac. It has long been thought that inner cells of the islands become blood cells, whereas the external ones develop into endothelium [69]. This, however, may not be the case. Detailed fluorescent immunohistochemical analysis of the early yolk sac revealed quite a different picture of extraembryonic mesoderm specification [70]. The proximal mesodermal cell masses turned out to specialize almost exclusively into blood cells. Apparently, the proximal yolk sac is the first anatomical site of active *de novo* hematopoiesis in the conceptus. Emerging more distally vascular plexus was found to expand proximally and envelop emerging blood cells squeezing them inside of the newly formed vessels. Massive red spots of blood islands probably correspond to big cell aggregations that become later vascularized and release blood cells into developing vascular plexus (Figure 1a). Some cell clusters still look quite dense even when yolk sac becomes well vascularized (Figure 1b). These transient aggregations may give rise to Runx1^high^ clusters of definitive precursor cells, which presumably contain members of the HSC lineage [6]. A great majority of cells arising from the proximal mesodermal masses are primitive erythroblasts, and they start to spread distally before blood circulation is initiated (Figure 1c). These cells might be pushed by the large number of blood island cells which become non-adherent at E8.0-E8.5. Circulation significantly increases the spreading, but for some period of time most of red blood cells are still retained within the yolk sac [71], which suggests that development of vasculature in the yolk sac substantially outpaces it in the embryo proper. Importantly, the cells with the definitive progenitor potential have significantly higher affinity to the yolk sac microenvironment at E9.5-E10.5 suggesting that the yolk sac works as temporary pre-liver niche for immature blood cells precursors [71]. Some of those precursors can be visualized by staining with CD41, and clusters of CD41^bright^ cells can be seen at the early somite stage within blood island [70]. It is possible that the CD41^bright^ clusters correspond to the Runx1^high^ cell aggregates detected at the same stage and in the same region by LacZ staining [6].

**Figure 2:**
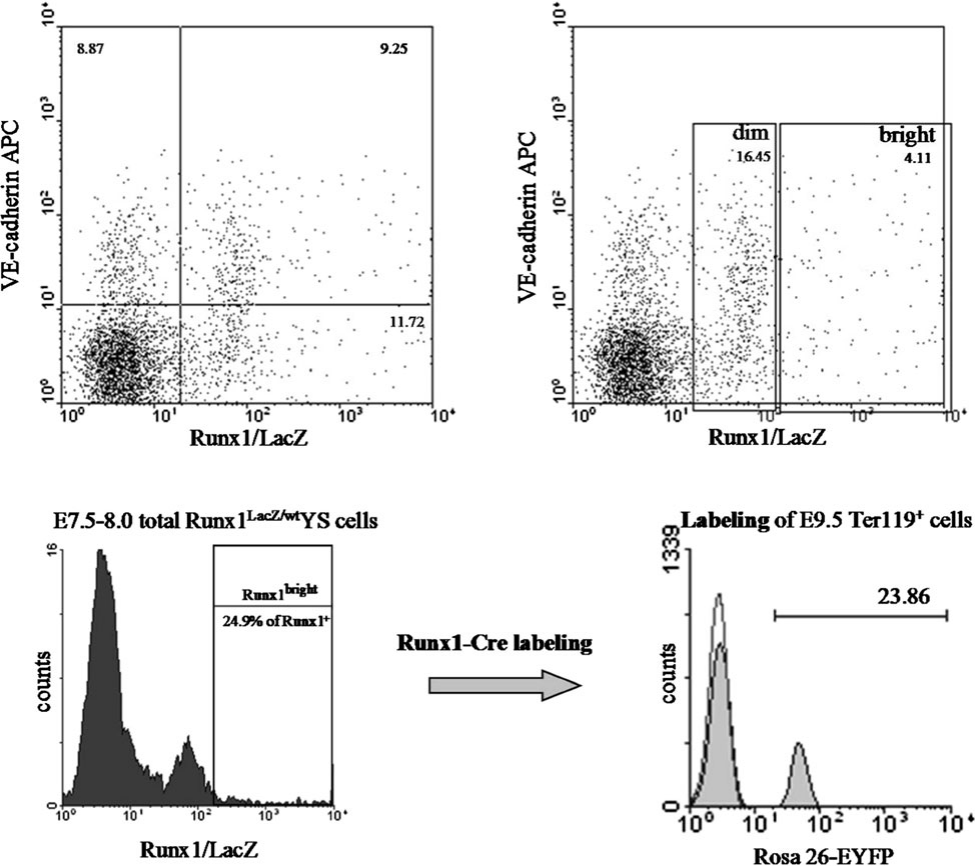
**Mesodermal cell masses in the proximal yolk sac co-express Runx1 and VE-cadherin.** Two upper panels show the flow cytometry analysis of pooled cells from eight E7.5-8.0 yolk sacs. Two lower panels demonstrate connection between the level of the Runx1 expression and the efficiency of cell labeling in the cell tracing studies [6].

The proximal mesodermal cell masses recently were shown to coexpress a number of hematopoietic and endothelial markers [8, 72]. Practically all Gata1- and majority of Runx1-positive cells in the mesodermal masses at the neural plate stage transiently coexpress VE-cadherin [8, 72, 73], the most specific marker of endothelial lineage. Gata1 and Runx1 are the key transcription factors for erythroid and entire definitive hematopoietic development, respectively [74, 75]. Slightly later at E7.75 (head hold stage), an embryonic globin marker of primitive erythroblasts is also transiently co-expressed with VE-cadherin apparently in the nascent embryonic red blood cells. It is unclear what function, if any, VE-cadherin is playing in the developing hematopoietic lineage, but the co-expression of the hematopoietic factors with VE-cadherin in non-endothelial cells suggests that unique epigenetic changes happen during *de novo* hematopoiesis. VE-cadherin^+^Runx1^+^ cells were found to be the earliest definitive hematopoietic precursors [8] and the co-expression is sustained for some period of time - about half of Runx1^+^ YS cells are still VE-cadherin-positive at the neural fold stage (Figure 2). Interestingly, only about 25% of all Runx1-positive cells at the neural plate can be arbitrarily qualified as Runx1^high^ (Figure 2) which underscores cell heterogeneity of the developing blood islands. It remains to be investigated whether the level of Runx1 expression has a functional significance, but the Runx1^high^ cells as preferable targets for Cre-dependent recombination in the Runx1-based cell tracing (Figure 2) eventually develop into adult hematopoietic cells including dHSCs [6, 8].

It is reasonable to assume that during hematopoietic specification the mesodermal masses are epigenetically distinct from both truly hematopoietic and endothelial cells as well as from “unspecified” or hemangioblast-like lateral mesoderm. The proximal yolk sac mesodermal masses therefore emerge as a unique cell population within developing conceptus. The endothelial marker expression at an early phase of mesoderm differentiation might reflect the default endothelial commitment of extraembryonic mesoderm [76], being a transient “leftover” from a more primitive developmental program [77], or it manifests yet unknown regulatory function of some endothelial markers in the hematopoietic development [78].

At about the same time the HSC lineage is being induced in the extraembryonic mesoderm cell masses [6, 8]. We do not know the characteristics of first cell belonging to the lineage, but it is tempting to see them as primitive phagocytic or macrophage-like cells. The early yolk sac was recently shown to be an independent source of microglia and skin dendritic cell lineages [7, 79], and it would not be surprising if other adult macrophage lineages arise autonomously in the extraembryonic mesoderm. Along with oxygen supply, the embryonic blood should also provide a significant number of phagocytes to clear up apoptotic cells in embryo [80, 81]. These phagocytes may emerge in blood islands as not fully functional primitive macrophages or macrophage precursors, and in contrast to the erythroblasts some of them could maintain or develop multipotentiality [82]. In this view, the intraaortic hemogenic clusters may reflect the process of macrophage extravasation [6] induced by such inflammatory factors as prostaglandin PGE2 [83], which have been shown to enhance the HSC generation in the AGM region of zebra fish and may also be able to do it in mammalian embryo [84]. Type II vasculogenesis [85] may be assisted by monocytes/macrophages as they are thought to be able to incorporate into endothelial layers [86, 87].

In addition to HSC lineage cells, the progeny of the yolk sac mesodermal masses also includes some emerging hematopoietic progenitors that have a limited self-renewal potential [88, 89]. These progenitors may be responsible for production of definitive erythrocytes beginning from E12.0 and also involved in the *in vivo* production of first definitive myeloid cells.

The inability of the yolk sac cells to demonstrate the dHSC potential can be attributed to the essential immaturity of early members of the HSC lineage that arise during *de novo* hematopoiesis. In order to understand the HSC development, more attention should be directed towards the proximal extraembryonic region of the visceral yolk sac. Learning about *in vivo* dHSC development will be undoubtedly instrumental in the design of efficient, reproducible and safe protocols for *in vitro* HSC generation.

## Conclusions

**Figure 3:**
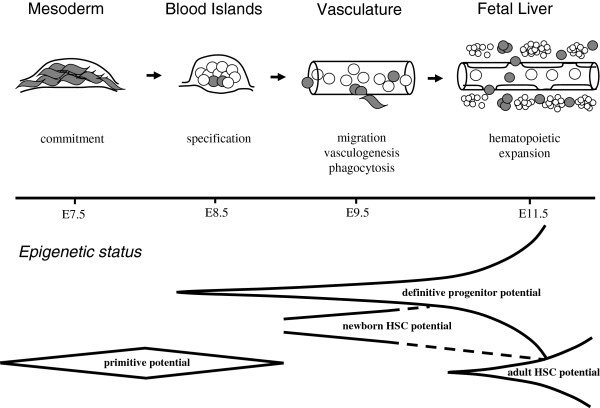
**Migration of blood cell precursors in the conceptus and the dynamics of their developmental potential.** Red cells are shown by open circles. Fenestrated endothelium of fetal liver sinusoids promotes the entry of hematopoietic progenitors into the first extravascular niche.

Current picture of the mammalian blood development was created using methods intended for studying adult hematopoiesis. It seems that most of controversies in developmental hematology arise from the excessive reliance on measuring cell potentials rather than cells fates. Unsettled epigenetic status of early embryonic cells makes them prone to show a variety of potentials which do not reflect the actual process of hematopoietic ontogenesis. Therefore, we can hardly attribute distinct hematopoietic phenotypes of adult hematology to hematopoietic precursors in the developing conceptus. Due to overbearing cell potential research, the role of the yolk sac in creation of HSC lineage was seriously underestimated by standard cell potential assays. As shown by the cell tracing studies, the proximal mesodermal masses of yolk sac may represent a unique cell population of the single hematopoietic origin in mammals. In the new emerging picture of hematopoietic ontogenesis the AGM region, umbilical/vitelline arteries and placental vasculature are not autonomous sources of definitive hematopoiesis. Instead, they operate as transient intravascular niches for developing HSC lineage (Figure 3). The first extravascular niche is formed within the fetal liver which functions as the first *bona fide* hematopoietic organ in mammalian ontogenesis.

The efficient protocol for generating the HSC potential *in vitro* is unlikely to be established empirically. It is hardly the way to translate our studies into safe and reproducible clinical applications. Accepting the pivotal role of yolk sac as the single origin of adult-type hematopoiesis is the key for developing the recapitulation strategy for efficient production of dHSC in well-defined artificial microenvironment.

## References

[1] Bowie MB, McKnight KD, Kent DG, McCaffrey L, Hoodless PA, Eaves CJ (2006). Hematopoietic stem cells proliferate until after birth and show a reversible phase-specific engraftment defect. J Clin Invest.

[2] Mikkola HKA, Orkin SH (2006). The journey of developing hematopoietic hematopoietic cells. Development.

[3] Medvinsky A, Rybtsov S, Taoudi S (2011). Embryonic origin of the adult hematopoietic system: advances and questions. Development.

[4] Cumano A, Godin I (2007). Ontogeny of the hematopoietic system. Annu Rev Immunol.

[5] McGrath KE, Palis J (2005). Hematopoiesis in the yolk sac: more than meets the eye. Exp Hematol.

[6] Samokhvalov IM, Samokhvalova NI, Nishikawa SI (2007). Cell tracing shows the contribution of the yolk sac to adult haematopoiesis. Nature.

[7] Ginhoux F, Greter M, Leboeuf M, Nandi S, See P, Gokhan S, Mehler MF, Conway SJ, Ng LG, Stanley ER, Samokhvalov IM, Merad M (2010). Fate mapping analysis reveals that adult microglia derive from primitive macrophages. Science.

[8] Tanaka Y, Hayashi M, Kubota Y, Nagai H, Sheng G, Nishikawa SI, Samokhvalov IM (2012). Early ontogenic origin of the hematopoietic stem cell lineage. Proc. Natl. Acad. Sci. USA.

[9] Zernicka-Goetz M (2005). Cleavage pattern and emerging asymmetry of the mouse embryo. Nat Rev Mol Cell Biol.

[10] Harris RG, Herzog EL, Bruscia EM, Grove JE, Van Arnam JS, Krause DS (2004). Lack of fusion requirement for development of bone marrow-derived epithelia. Science.

[11] Anderson PA, Müller-Borer BJ, Esch GL, Coleman WB, Grisham JW, Malouf NN (2007). Calcium signals induce liver stem cells to acquire a cardiac phenotype. Cell Cycle.

[12] Shefer G, Wleklinski-Lee M, Yablonka-Reuveni Z (2004). Skeletal muscle satellite cells can spontaneously enter an alternative mesenchymal pathway. J Cell Sci.

[13] Wurmser AE, Nakashima K, Summers RG, Toni N, D’Amour KA, Lie DC, Gage FH (2004). Cell fusion-independent differentiation of neural stem cells to the endothelial lineage. Nature.

[14] Takahashi K, Yamanaka S (2006). Induction of pluripotent stem cells from mouse embryonic and adult fibroblast cultures by defined factors. Cell.

[15] Okita K, Ichisaka T, Yamanaka S (2007). Generation of germline-competent induced pluripotent stem cells. Nature.

[16] Wernig M, Meissner A, Foreman R, Brambrink T, Ku M, Hochedlinger K, Bernstein BE, Jaenisch R (2007). ***In vitro*** reprogramming of fibroblasts into a pluripotent ES-cell-like state. Nature.

[17] Gilmore AP (2005). Anoikis. Cell Death Differ.

[18] Müller AM, Medvinsky AL, Strouboulis J, Grosveld F, Dzierzak E (1994). Development of hematopoietic stem cell activity in the mouse embryo. Immunity.

[19] Godin I, Garcia-Porrero JA, Dieterlen-Lievre F, Cumano A (1999). Stem cell emergence and hemopoietic activity are incompatible in mouse intraembryonic sites. J Exp Med.

[20] Palis J, Robertson S, Kennedy M, Wall C, Keller G (1999). Development of erythroid and myeloid progenitors in the yolk sac and embryo proper of the mouse. Development.

[21] Minot CS, Keibel F, Mall FP (1912). Development of the blood, the vascular system and the spleen. Manual of Human Embryology.

[22] Dzierzak E, Speck NA (2008). Of lineage and legacy: the development of mammalian hematopoietic stem cells. Nat Immunol.

[23] Pimanda JE, Ottersbach K, Knezevic K, Kinston S, Chan WYI, Wilson NK, Landry JR, Wood AD, Kolb-Kokocinsky A, Green AR, Tannahill D, Lacaud G, Kouskoff V, Göttgens B (2007). Gata2, Fli1, and Scl form a recursively wired gene-regulatory circuit during early hematopoietic development. Proc. Natl. Acad. Sci. USA.

[24] Morrison SJ, Wandycz AM, Akashi K, Globerson A, Weissman IL (1996). The aging of hematopoietic stem cells. Nat Med.

[25] Akashi K, Traver D, Miyamoto T, Weissman IL (2000). A clonogenic common myeloid progenitor that gives rise to all myeloid lineages. Nature.

[26] Yilmaz ÖH, Valdez R, Theisen BK, Guo W, Ferguson DO, Wu H, Morrison SJ (2006). *Pten* dependence distinguishes haematopoietic stem cells from leukaemia-initiating cells. Nature.

[27] Kiel MJ, Radice GL, Morrison SJ (2007). Lack of evidence that hematopoietic stem cells depend on N-cadherin-mediated adhesion to osteoblasts for their maintenance. Cell Stem Cell.

[28] Sudo K, Ema H, Morita Y, Nakauchi H (2000). Age-associated characteristics of murine hematopoietic stem cells. J Exp Med.

[29] Yoder MC, Hiatt K, Mukherjee P (1997). *In vivo* repopulating hematopoietic stem cells are present in the murine yolk sac at day 9.0 postcoitus. Proc. Natl. Acad. Sci. USA.

[30] Yoder MC, Hiatt K, Dutt P, Mukherjee P, Bodine DM, Orlic D (1997). Characterization of definitive lymphohematopoietic stem cells in the day 9 murine yolk sac. Immunity.

[31] Matsuoka S, Tsuji K, Hisakawa H, Xu M, Ebihara Y, Ishii T, Sugiyama D, Manabe A, Tanaka R, Ikeda Y, Asano S, Nakahata T (2001). Generation of definitive hematopoietic stem cells from murine early yolk sac and paraaortic splanchnopleures by aorta-gonad-mesonephros region–derived stromal cells. Blood.

[32] Cumano A, Dieterlen-Lievre F, Godin I (1996). Lymphoid potential, probed before circulation in mouse, is restricted to caudal intraembryonic splanchnopleura. Cell.

[33] Cumano A, Ferraz JC, Klaine M, Di Santo JP, Godin I (2001). Intraembryonic, but not yolk sac hematopoietic precursors, isolated before circulation, provide long-term multilineage reconstitution. Immunity.

[34] Huber TL, Kouskoff V, Fehling HJ, Palis J, Keller G (2004). Haemangioblast commitment is initiated in the primitive streak of the mouse embryo. Nature.

[35] Ferkowicz MJ, Yoder MC (2005). Blood island formation: longstanding observations and modern interpretations. Exp Hematol.

[36] Morrison SJ, Wright DE, Cheshier SH, Weissman IL (1997). Hematopoietic stem cells: challenges to expectations. Curr Opin Immunol.

[37] Kinder SJ, Tsang TE, Quinlan GA, Hadjantonakis AK, Nagy A, Tam PPL (1999). The orderly allocation of mesodermal cells to extraembryonic structures and the anteroposterior axis during gastrulation of the mouse embryo. Development.

[38] Ueno H, Weissman IL (2006). Clonal analysis of mouse development reveals a polyclonal origin for yolk sac blood islands. Dev Cell.

[39] Sumner R, Crawford A, Mucenski M, Frampton J (2000). Initiation of adult myelopoiesis can occur in the absence of c-Myb whereas subsequent development is strictly dependent on the transcription factor. Oncogene.

[40] Kaufman MH (1992). The atlas of mouse development.

[41] Kingsley PD, Malik J, Fantauzzo KA, Palis J (2004). Yolk sac-derived primitive erythroblasts enucleate during mammalian embryogenesis. Blood.

[42] Speck N, Peeters M, Dzierzak E, Rossant J, Tam PPL (2002). Development of the vertebrate hematopoietic system. Mouse development. Patterning, morphogenesis, and organogenesis.

[43] Jaffredo T, Nottingham W, Liddiard K, Bollerot K, Pouget C, de Bruijn M (2005). From hemangioblast to hematopoietic stem cell: an endothelial connection?. Exp Hematol.

[44] Nishikawa SI, Nishikawa S, Kawamoto H, Yoshida H, Kizumoto M, Kataoka H, Katsura Y (1998). In vitro generation of lymphohematopoietic cells from endothelial cells purified from murine embryos. Immunity.

[45] Hirai H, Ogawa M, Suzuki N, Yamamoto M, Breier G, Mazda O, Imanishi J, Nishikawa SI (2003). Hemogenic and nonhemogenic endothelium can be distinguished by the activity of fetal liver kinase (Flk)-1 promoter/enhancer during mouse embryogenesis. Blood.

[46] North TE, de Bruijn MF, Stacy T, Talebian L, Lind E, Robin C, Binder M, Dzierzak E, Speck NA (2002). Runx1 expression marks long-term repopulating hematopoietic stem cells in the midgestation mouse embryo. Immunity.

[47] Betrand JY, Giroux S, Golub R, Klaine M, Jalil A, Boucontet L, Godin I, Cumano A (2005). Characterization of purified intraembryonic hematopoietic stem cells as a tool to define their site of origin. Proc. Natl. Acad. Sci. USA.

[48] Walls JR, Coultas L, Rossant J, Henkelman RM (2008). Three-dimensional analysis of vascular development in the mouse embryo. PLoS One.

[49] North T, Gu TL, Stacy T, Wang Q, Howard L, Binder M, Marin-Padilla M, Speck NA (1999). Cbfa2 is required for the formation of the intra-aortic hematopoietic clusters. Development.

[50] Kissa K, Herbomel P (2010). Blood stem cells emerge from aortic endothelium by a novel type of cell transition. Nature.

[51] Starz-Gaiano M, Lehmann R (2001). Moving towards the next generation. Mech Dev.

[52] Anderson R, Copeland TK, Schöler H, Heasman J, Wylie C (2000). The onset of germ cell migration in the mouse embryo. Mech Dev.

[53] Rich IN (1995). Primordial germ cells are capable of producing cells of the hematopoietic system *in vitro*. Blood.

[54] Ohtaka T, Matsui Y, Obinata M (1999). Hematopoietic development of primordial germ cell-derived mouse embryonic germ cells in culture. Biochem Biophys Res Commun.

[55] Wylie C, Anderson R, Rossant J, Tam PPL (2002). Germ cells. Mouse development. Patterning, morphogenesis, and organogenesis.

[56] Wakayama T, Hamada K, Yamamoto M, Suda T, Iseki S (2003). The expression of platelet endothelial cell adhesion molecule-1 in mouse primordial germ cells during their migration and early gonadal formation. Histochem Cell Biol.

[57] De Felici M, Scaldaferri ML, Farini D (2005). Adhesion molecules for mouse primordial germ cells. Front Biosci.

[58] Anderson R, Fassler R, Georges-Labouesse E, Hynes RO, Bader BL, Kreidberg JA, Schaible K, Heasman J, Wylie C (1999). Mouse primordial germ cells lacking beta1 integrins enter the germline but fail to migrate normally to the gonads. Development.

[59] Ara T, Nakamura Y, Egawa T, Sugiyama T, Abe K, Kishimoto T, Matsui Y, Nagasawa T (2003). Impaired colonization of the gonads by primordial germ cells in mice lacking a chemokine, stromal cell-derived factor-1 (SDF-1). Proc. Natl. Acad. Sci. USA.

[60] Gofflot F, Hall M, Morriss-Kay GM (1997). Genetic patterning of the developing mouse tail at the time of posterior neuropore closure. Dev Dyn.

[61] Beddington RSP (1981). An autoradiographic analysis of the potency of embryonic ectoderm in the 8th day postimplantation mouse embryo. J Embryol Exp Morphol.

[62] Beddington RPS (1982). An autoradiographic analysis of tissue potency in different regions of the embryonic ectoderm during gastrulation in the mouse. J Embryol Exp Morphol.

[63] Medvinsky A, Dzierzak E (1996). Definitive hematopoiesis is autonomously initiated by the AGM region. Cell.

[64] Cai Z, de Bruijn M, Ma X, Dortland B, Luteijn T, Downing JR, Dzierzak E (2000). Haploinsufficiency of AML1 affects the temporal and spatial generation of hematopoietic stem cells in the mouse embryo. Immunity.

[65] Wood HB, May G, Healy L, Enver T, Morriss-Kay GM (1997). CD34 expression patterns during early mouse development are related to modes of blood vessel formation and reveal additional sites of hematopoiesis. Blood.

[66] de Bruijn M, Speck NA, Peeters MC, Dzierzak E (2000). Definitive hematopoietic stem cells first develop within the major arterial regions of the mouse embryo. EMBO J.

[67] Corbel C, Salaün J (2002). αIIb integrin expression during development of the murine hematopoietic system. Dev Biol.

[68] Gardner RL, Rossant J, Tam PPL (2002). Asymmetry and prepattern in mammalian development. Mouse development. Patterning, morphogenesis, and organogenesis.

[69] Maximow AA (1924). Relation of blood cells to connective tissues and endothelium. Physiol Rev.

[70] Ferkowicz MJ, Starr M, Xie X, Li W, Johnson SA, Shelley WC, Morrison PR, Yoder MC (2003). CD41 expression defines the onset of primitive and definitive hematopoiesis in the murine embryo. Development.

[71] McGrath KE, Koniski AD, Malik J, Palis J (2003). Circulation is established in a stepwise pattern in the mammalian embryo. Blood.

[72] Ema M, Yokomizo T, Wakamatsu A, Terunuma T, Yamamoto M, Takahashi S (2006). Primitive erythropoiesis from mesodermal precursors expressing VE-cadherin, PECAM-1, Tie2, endoglin, and CD34 in the mouse embryo. Blood.

[73] Yokomizo T, Takahashi S, Mochizuki N, Kuroha T, Ema M, Wakamatsu A, Shimizu S, Ohneda O, Osato M, Okada H, Komori T, Ogawa M, Nishikawa SI, Ito Y, Yamamoto M (2007). Characterization of GATA-1^+^ hemangioblastic cells in the mouse embryo. EMBO J.

[74] Orkin S (1992). GATA-binding transcription factors in hematopoietic cells. Blood.

[75] Okuda T, Van Deursen J, Hiebert SW, Grosveld G, Downing JR (1996). AML1, the target of multiple chromosomal translocations in human leukemia, is essential for normal fetal liver hematopoiesis. Cell.

[76] Samokhvalov IM (2012). A long way to stemness. Cell Cycle.

[77] Kim I, Yilmaz OH, Morrison SJ (2005). CD144 (VE-cadherin) is transiently expressed by fetal liver hematopoietic stem cells. Blood.

[78] Dejana E, Bazzoni G, Lampugnani MG (1999). Vascular Endothelial (VE)-cadherin: only an intercellular glue?. Exp Cell Res.

[79] Hoeffel G, Wang Y, Greter M, See P, Teo P, Malleret B, Leboeuf M, Low D, Oller G, Almeida F, Choy SHY, Grisotto M, Renia L, Conway SJ, Stanley ER, Chan JKY, Ng LG, Samokhvalov IM, Merad M, Ginhoux F (2012). Adult Langerhans cells derive predominantly from embryonic fetal liver monocytes with a minor contribution of yolk sac–derived macrophages. J Exp Med.

[80] Baehrecke EH (2002). How death shapes life during development. Nat Rev Mol Cell Biol.

[81] Lichanska AM, Hume DA (2000). Origins and functions of phagocytes in the embryo. Exp Hematol.

[82] Zhao Y, Glesne D, Huberman E (2003). A human peripheral blood monocytes-derived subset acts as pluripotent stem cells. Proc. Natl. Acad. Sci. USA.

[83] Nathan C (2002). Points of control in inflammation. Nature.

[84] North TE, Goessling W, Walkley CR, Lengerke C, Kopani KR, Lord AM, Weber GJ, Bowman TV, Jang IH, Grosser T, FitzGerald GA, Daley GQ, Orkin SH, Zon LI (2007). Prostaglandin E2 regulates vertebrate haematopoietic stem cell homeostasis. Nature.

[85] Poole TJ, Finkelstein EB, Cox CM (2001). The role of FGF and VEGF in angioblast induction and migration during vascular development. Dev Dyn.

[86] Fujiyama S, Amano K, Uehira K, Yoshida M, Nishiwaki Y, Nozawa Y, Jin D, Takai S, Miyazaki M, Egashira K, Imada T, Iwasaka T, Matsubara H (2003). Bone marrow monocyte lineage cell adhere on injured endothelium in a monocytes chemoattractant protein-1 – dependent manner and accelerate reendothelization as endothelial progenitor cells. Circ Res.

[87] Prater DN, Case J, Ingram DA, Yoder MC (2007). Working hypothesis to redefine endothelial progenitor cells. Leukemia.

[88] Emambokus NR, Frampton J (2003). The glycoprotein IIb molecule is expressed on early murine hematopoietic progenitors and regulates their numbers in sites of hematopoiesis. Immunity.

[89] Li Z, Godinho FJ, Klusmann JH, Garriga-Canut M, Yu C, Orkin SH (2005). Developmental stage-selective effect of somatically mutated leukomogenic transcription factor GATA1. Nat Genet.

